# Nonlinear Parameter Identification of a Resonant Electrostatic MEMS Actuator

**DOI:** 10.3390/s17051121

**Published:** 2017-05-13

**Authors:** Majed S. Al-Ghamdi, Ayman M. Alneamy, Sangtak Park, Beichen Li, Mahmoud E. Khater, Eihab M. Abdel-Rahman, Glenn R. Heppler, Mustafa Yavuz

**Affiliations:** 1 Department of Systems Design Engineering, University of Waterloo, Waterloo, ON N2L 3G1, Canada; malghamd@uwaterloo.ca (M.S.A.-G.); aalneamy@uwaterloo.ca (A.M.A.); sangtak@uwaterloo.ca (S.P.); heppler@uwaterloo.ca (G.R.H.); 2 Department of Mechanical and Mechatronics Engineering, University of Waterloo, Waterloo, ON N2L 3G1, Canada; myavuz@uwaterloo.ca; b84li@uwaterloo.ca (B.L.); myavuz@uwaterloo.ca (M.Y.); 3 Department of Mechanical Engineering, King Fahd University of Petroleum and Minerals, Dhahran 31261, Saudi Arabia; mkhater@kfupm.edu.sa

**Keywords:** electrostatic MEMS, primary resonance, secondary resonances, parameter identification, process noise, measurement noise

## Abstract

We experimentally investigate the primary superharmonic of order two and subharmonic of order one-half resonances of an electrostatic MEMS actuator under direct excitation. We identify the parameters of a one degree of freedom (1-DOF) generalized Duffing oscillator model representing it. The experiments were conducted in soft vacuum to reduce squeeze-film damping, and the actuator response was measured optically using a laser vibrometer. The predictions of the identified model were found to be in close agreement with the experimental results. We also identified the noise spectral density of process (actuation voltage) and measurement noise.

## 1. Introduction

MEMS products are dominating many engineering fields as the technology for micro fabrication continues to develop. Actuation and sensing stand as the core applications of MEMS in everyday life. MEMS actuators include MEMS switches which have been implemented in antenna switches, phase shifters, tunable filters, and many other high-frequency applications [1,2,3,4]. Other applications include micromirrors, clocks, and filters [5,6,7,8,9]. In all of these applications, electrostatic MEMS offer the advantages of elevated actuation density, relatively ‘large’ motions, low power consumption, and small footprint.

Electrostatic MEMS actuators encounter several nonlinearities. These include electrical and mechanical quadratic and cubic nonlinearities in addition to a pull-in instability. It is important to accurately account for these nonlinearities when designing MEMS actuators. This ensures an accurate prediction of the onset of bifurcations and instabilities as well as desirable device performance. To improve the performance of MEMS actuators, they are frequently operating at resonance. This can be done via different drive conditions, including primary, superharmonic, and subharmonic excitation. The frequency of the input excitation is tuned to be near the natural frequency of the actuator in the case of primary resonance, while it is tuned to be near half and twice the natural frequency for superharmonic and subharmonic excitations, respectively [10,11,12,13].

Younis and Nayfeh [14] analytically investigated the primary resonance of electrostatic MEMS actuators. Secondary resonances also play an important role in the actuation of MEMS. Abdel-Rahman and Nayfeh [15] theoretically predicted that exciting an electrostatic actuator near its subharmonic or superharmonic resonances will result in dynamic response on the same order-of-magnitude as primary resonance. Primary, superharmonic, and subharmonic resonances of an electrostatic actuator were demonstrated experimentally by [16].

Common types of noise that affect electrostatic actuators include electrical noise, thermo-mechanical (Brownian) noise, and environmental noise (external disturbances) [17,18,19]. Noise is a stochastic process that does not have a deterministic value in the time or frequency domains. However, it can be described by its power spectral density (PSD) measured over a wide frequency range [19]. The impact of electrical noise on electrostatic actuators is particularly prominent because it causes stochastic variations in the actuation voltage, representing process noise, and the measured output signal, representing measurement noise. The dominant sources of electrical noise are thermal noise due to Brownian motion and flicker noise due to random charge hold and release of events between Silicon dioxide (SiO${}_{2}$) and Silicon (Si) layers. Flicker noise (1/f) dominates electrical noise at low frequencies, while thermal noise dominates at high frequencies [17,19,20].

In this paper, we present a process to identify a nonlinear model for electrostatic MEMS actuators undergoing primary, superharmonic, and subharmonic excitations, as well as process and measurement noise. The parameter identification process exploits experimentally-obtained Fast Fourier Transforms (FFTs) of the actuator response under excitations in the vicinity of those resonances. The velocity of a representative point on the actuator in response to those excitations is measured optically using a laser Doppler vibrometer (LDV).

## 2. Model and Experiment

**Figure 1:**
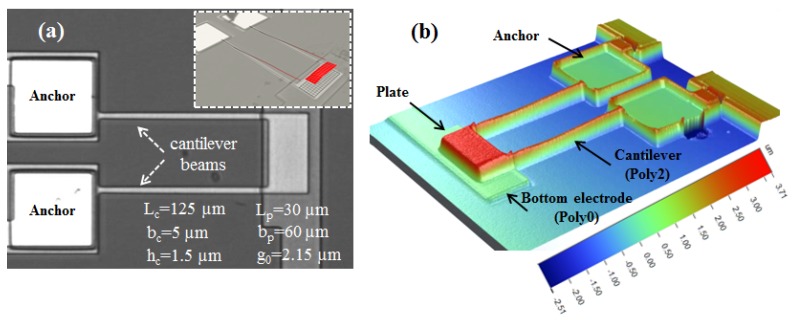
Pictures of the actuator under (**a**) the microscope and (**b**) white light profilometer. Inset: vibrometer multi-scan points showing the actuator response.

The actuator is fabricated from polysilicon using the Poly2 layer in the PolyMUMPs fabrication process [21]. It features two support beams with the nominal (design) dimensions 125μm ×5μm ×1.5μm and an end microplate with the nominal dimensions 30μm ×60μm ×1.5μm, Figure 1a. The beams are attached at the plate edges to maximize the torsional stiffness. Two gold pads are patterned at the roots of the support beams to apply a potential difference between the plate and a bottom electrode. A 3-D scan of the actuator using a white light profilometer is shown in Figure 1b.

**Figure 2:**
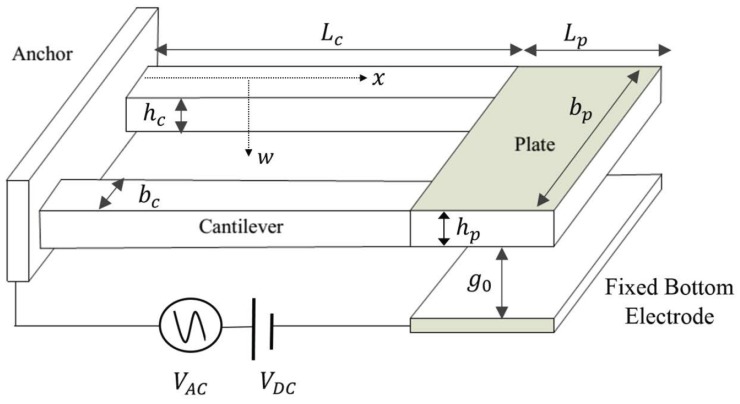
Schematic of the actuator.

The plate is electrostatically excited by applying a voltage difference

(1) $$V\left(t\right)=V_{DC}+V_{AC}\cos\left(\Omega t\right)$$

between its microplate and a fixed bottom electrode, Figure 2. The nominal capacitive gap between the plate and the fixed bottom electrode is $g_{\circ }=2.15\;\mu$m. The actuator is modeled as a single-degree-of-freedom lumped model in which the equation governing the transverse plate displacement w(t) is given by [14,15]:

(2) $$m\ddot{w}+c\dot{w}+k_{1}w+k_{2}w^{2}+k_{3}w^{3}=\frac{\epsilon AV^{2}}{2{\left(g_{\circ }-w\right)}^{2}}$$

where *c* is the viscous damping coefficient; ε is air permittivity, and *A* is the plate area. The effective mass of the actuator is found as [22]

(3) $$m=\rho (L_{p}b_{p}h_{P}+0.46L_{c}b_{c}h_{c})$$

where ρ=2300 kg/m${}^{3}$ is the density of polysilicon. The linear, quadratic and cubic stiffness coefficients are denoted by $k_{1}$, $k_{2}$, and $k_{3}$, respectively. The inclusion of quadratic and cubic stiffnesses is meant to enhance the lumped model by accounting for inherent nonlinearities.

With the assumptions that the cantilever beams are identical, the microplate is rigid, and the distributed electrostatic force is lumped at the center of the microplate, the boundary conditions at the end of the support beams can be described as a combined electrostatic shear and moment. The linear stiffness of the beams can then be written as:

(4) $$k_{1}=\frac{2Eb_{c}h_{c}^{3}}{4L_{c}^{3}+3L_{c}^{2}L_{p}}$$

where E=160 GPa is Young’s modulus for polysilicon; $L_{p}$, $L_{c}$, $b_{c}$ and $h_{c}$ are the plate length, individual beam length, beam width, and beam thickness, respectively.

Two sources of noise were introduced into the model: process noise and measurement noise. Process noise was represented by a white noise term added to the actuation signal in the frequency domain

(5) $$\hat{V}\left(f\right)=V\left(f\right)+S_{v}\sqrt{BW_{v}}$$

where $S_{v}$ is the noise spectral density and $\left(BW_{v}=\Omega \right)$ is the bandwidth of the actuation signal. Measurement noise was represented by another white noise term added to the predicted velocity in the frequency domain

(6) $$\hat{\dot{w}}\left(f\right)=\dot{w}\left(f\right)+S_{m}\sqrt{BW_{m}}$$

where $\hat{\dot{w}}\left(f\right)$ is the predicted velocity with measurement noise, $S_{m}$ is the noise spectral density and $BW_{m}$ is the bandwidth of the measurement signal. The laser Doppler vibrometer sampling rate was set to 256 ksamples/s and the bandwidth to $BW_{m}=100$ kHz.

The actuator was placed in a vacuum chamber (pressure 48 mTorr) to reduce the effect of squeeze-film damping, thereby increasing its quality factor. The excitation voltage was supplied via an electrical feed-through. The velocity of the microplate center point was measured using the VD-02 velocity decoder of the vibrometer.

First, a low-frequency (<1 kHz) pulse train was applied to the actuator. The FFT of the plate velocity was measured and used to identify the fundamental natural frequency $\omega_{n}=32.8$ kHz. Next, the actuator dynamic response was investigated under primary, subharmonic, and superharmonic excitations. The excitation signal (Equation 1) was set to an amplitude of $V_{DC}=500$ mV and $V_{AC}=500$ mV and the frequencies $\Omega =\omega_{n}$, $\Omega =\frac{1}{2}\omega_{n}$, and $\Omega =2\omega_{n}$, successively. The experimental results in each case were compared to the steady-state response of the model obtained numerically by integrating Equation (2) for 4000T, where T=2π/Ω is the excitation period. A parameter identification procedure was developed to estimate the actuator dimensions such that the differences between the numerical and experimental steady-state responses were minimized.

## 3. Primary Resonance

**Figure 3:**
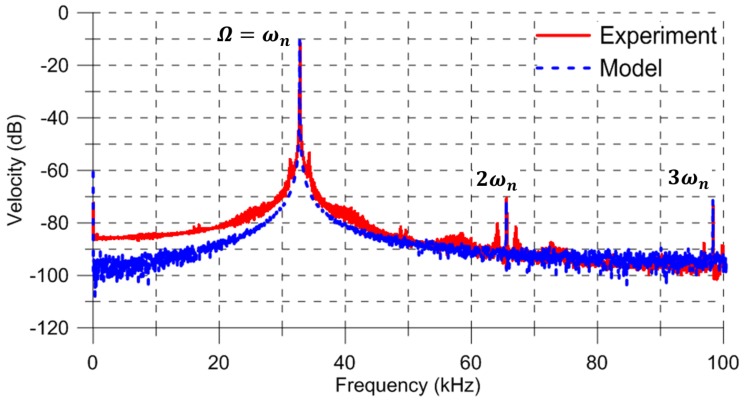
The experimental (red solid line) and model predicted (blue dashed line) FFTs of the actuator velocity under a primary resonant excitation $\Omega =\omega_{n}$ (0 dB= 1 m/s).

As a first step towards understanding the system dynamics, it was excited in the vicinity of primary resonance of the first out-of-plane bending mode. The experimentally-determined shape of this mode is shown in the inset of Figure 1a. The excitation frequency was set to Ω=32.8 kHz, and the FFT of the center-point velocity was calculated using the vibrometer’s software [23]—it is shown by the red line in Figure 3. The maximum measured velocity was 304.15 mm/s, corresponding to a displacement of 1.47 μm.

The measurement noise spectral density $S_{m}$ was calculated from the FFT using the formula

(7) $$S_{m}=\frac{1}{q-p+1}\sum_{i=p}^{q}\hat{\dot{w}}\left(f\right)$$

where $\hat{\dot{w}}\left(f\right)$ is velocity with measurement noise in dB-scale (0 dB= 1 m/s) and *p* and *q* are the FFT bin numbers limiting the region in the frequency spectrum over which the average noise spectral density is obtained. Using a frequency range away from the excitation frequency Ω and its harmonics at 2Ω and 3Ω, we calculated $S_{m}=21.72$ (μm/s)$/\sqrt{\mathrm{Hz}}$ over the frequency range [68,80] kHz. It can be observed in Figure 3 that measurement noise dominates the response in this frequency range.

The dominant peak of the FFT was found at the fundamental natural frequency of the actuator $\omega_{n}=32.8$ kHz. Equations (3) and (4) were used to substitute in the linear natural frequency equation

(8) $$\omega_{n}=\sqrt{\frac{k_{1}}{m}}$$

and its measured value was used to estimate the beam width and the structural layer thickness as $b_{c}=4.4\;\mu$m and $h_{c}=1.15\;\mu$m. The mass and linear stiffness were calculated from Equations (3) and (4) as m=5.5 ng and $k_{1}=0.2305$ N/m, respectively.

Three distinct peaks are observed in the FFT at the first, second, and third harmonics of the excitation signal $\omega_{n}$, $2\omega_{n}$, and $3\omega_{n}$, respectively. The values of the quadratic and cubic nonlinearities were estimated by matching the locations of the second and third harmonic peaks in the model-predicted FFT to that obtained experimentally. They were found to be $k_{2}=0.046$ N/m${}^{2}$ and $k_{3}=0.059$ N/m${}^{3}$. The capacitive gap was maintained in the identification process at its nominal value $g_{\circ }=2.15\;\mu$m.

The FFT of the numerically predicted velocity with the measurement noise $\hat{\dot{w}}$ was obtained for the last 400 excitation periods, as shown by the blue dashed line in Figure 3. The placement of the actuator inside the vacuum chamber elevated its quality factor, which was estimated as Q=1300 by matching the half-power bandwidth of the peaks in the experimental and numerical FFTs (Figure 3). Typically, the actuator settles down to steady-state response within a time period $QT_{n}$, where $T_{n}$ is the natural period. Our estimate of the quality factor is therefore consistent with the use of long-time integration over a time horizon of 4000T to obtain the steady-state response.

To match the peak values of experimental and numerical FFTs, the process noise spectral density was set to $S_{v}=$ 0.063 V$/\sqrt{\mathrm{Hz}}$ over a bandwidth of $BW_{v}=32.8$ kHz. The measurement noise spectral density was calculated as $S_{m}=21.89$ (μm/s)$/\sqrt{\mathrm{Hz}}$ by applying Equation (7) to the numerically predicted FFT over the frequency range [68,80] kHz. We note that the addition of measurement noise to the model allowed the predicted FFT to match the skirt of the peaks at $\omega_{n}$, $2\omega_{n}$, and $3\omega_{n}$ and the noise floor of the experimental FFT.

The close agreement between the experimental and model-predicted FFTs in the vicinity of the peaks indicates that a generalized Duffing oscillator model, process noise, and the proposed identification procedure are adequate to capture the large-amplitude motions of the electrostatic actuator. On the other hand, it is necessary to include measurement noise in the model to capture the small-amplitude motions (away from resonances) of the actuator.

## 4. Superharmonic Resonance

**Figure 4:**
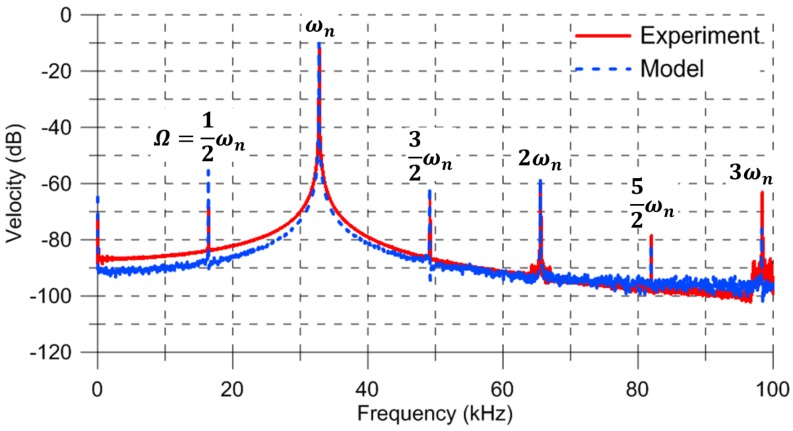
The experimental (red solid line) and model predicted (blue dashed line) FFTs of the actuator velocity under a superharmonic excitation $\Omega =\frac{1}{2}\omega_{n}$ (0 dB = 1 m/s).

The same experimental procedure as used previously was employed to investigate superharmonic resonance with the excitation frequency set to Ω=16.4 kHz. The FFT of the microplate center velocity obtained using the vibrometer is shown by the red line in Figure 4. The maximum measured velocity was 313.14 mm/s, corresponding to a displacement of 1.51 μm. The response demonstrates superharmonic resonance of order two with peaks at $\frac{1}{2}\omega_{n}$, $\omega_{n}$, $\frac{3}{2}\omega_{n}$, $2\omega_{n}$, $\frac{5}{2}\omega_{n}$, and $3\omega_{n}$. The measurement noise spectral density was calculated as $S_{m}=16.54$ (μm/s)$/\sqrt{\mathrm{Hz}}$ over the frequency range of [68,80] kHz.

The model parameters and numerical procedure described previously were used to evaluate the FFT of the velocity $\hat{\dot{w}}$ for the last 400 excitation periods—it is shown by the blue dashed line in Figure 4. To match the peak values of the predicted FFT to the experimental FFT, the process noise spectral density was set in the model to $S_{v}=$ 0.064 V$/\sqrt{\mathrm{Hz}}$ over a bandwidth of $BW_{v}=16.4$ kHz. Further, The measurement noise spectral density was calculated from Equation (7) as $S_{m}=18.89$ (μm/s)$/\sqrt{\mathrm{Hz}}$ over the frequency range [68,80] kHz. Close agreement is observed between the experimental and numerical predicted FFTs, except for the peak at $\Omega =\frac{1}{2}\omega_{n}$.

## 5. Subharmonic Resonance

**Figure 5:**
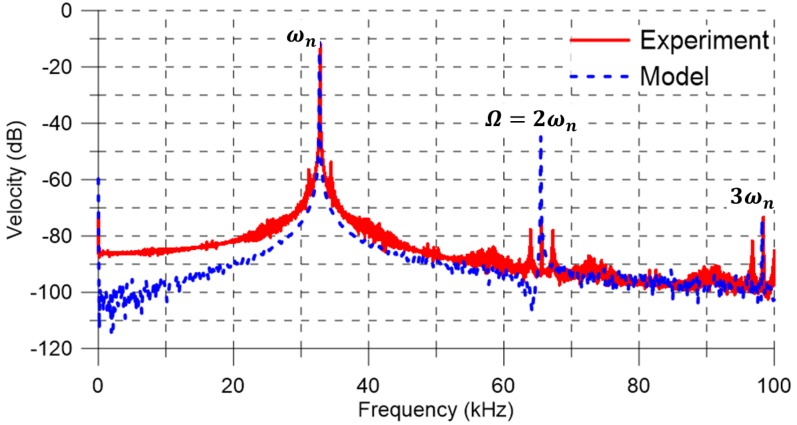
The experimental (red solid line) and model-predicted (blue dashed line) FFTs of the actuator velocity under a subharmonic excitation $\Omega =2\omega_{n}$ (0 dB = 1 m/s).

The experimental procedure was repeated for subharmonic resonance under an excitation frequency of Ω=65.6 kHz. The FFT of the microplate center velocity was obtained optically using the vibrometer, and is shown by the red line in Figure 5. The maximum measured velocity was 311.85 mm/s, corresponding to a displacement of 1.51 μm. The response demonstrates a typical subharmonic resonance of order one-half with peaks at $\omega_{n}$, $2\omega_{n}$, and $3\omega_{n}$. In addition, peaks are also observed at $\frac{1}{2}\omega_{n}$, $\frac{3}{2}\omega_{n}$, and $\frac{5}{2}\omega_{n}$. We estimated the measurement noise spectral density using Equation (7) over the frequency range of [68,80] kHz as $S_{m}=20.22$ (μm/s)$/\sqrt{\mathrm{Hz}}$.

The values of the quadratic and cubic nonlinearities were reduced to $k_{2}=0.324$ N/m${}^{2}$ and $k_{3}=0.199$ N/m${}^{3}$ in order to match the peaks of the numerical and experimental FFTs. The numerically-predicted FFT is shown by the blue dashed line in Figure 5. The numerical procedure and the rest of the model parameters were unchanged. We note that the impact of the quadratic nonlinearity on the resonant peak at $\omega_{n}$ was more prominent compared that of the cubic nonlinearity.

To match the peak values of experimental and predicted FFTs, the process noise spectral density was set in the model to $S_{v}=$ 0.063 V$/\sqrt{\mathrm{Hz}}$ over a bandwidth of $BW_{v}=65.6$ kHz. The measurement noise spectral density was calculated from the predicted FFT over the frequency range [68,80] kHz using Equation (7) as $S_{m}=18.89$ (μm/s)$/\sqrt{\mathrm{Hz}}$. A good match is achieved between the model and experimental FFTs at the integer harmonics $\omega_{n}$, $2\omega_{n}$, and $3\omega_{n}$, but the harmonics at $\frac{1}{2}\omega_{n}$, $\frac{3}{2}\omega_{n}$, and $\frac{5}{2}\omega_{n}$ are absent from the model responses.

## 6. Conclusions

We presented a parameter identification technique to identify the lumped system parameters of an electrostatic MEMS actuator from experimentally-obtained FFTs of its responses in primary, superharmonic, or subharmonic resonances. Our results show that a generalized Duffing oscillator model in combination with process and measurement noise can accurately capture the motions and resonances of electrostatic actuators.

The FFTs of the measured velocity were obtained using a laser Doppler vibrometer. All the experiments were conducted in soft vacuum in order to reduce squeeze-film damping, and thereby elevate the actuator’s quality factor. The spectral density of measurement noise in the experimental FFT matched well with that in the model-predicted FFT, which demonstrates the model fidelity. In addition, our parameter identification technique was able to estimate the spectral density of process noise, which is difficult to measure experimentally.

The quadratic and cubic nonlinearities in the lumped mass model capture the effective nonlinearity, averaged over the response cycle due to the electrostatic field, mid-plane stretching, and other sources. These averages vary as the response cycle changes shape among the three excitation cases, as evidenced by variation in the relative power of the harmonics, thereby requiring independent identification of the effective nonlinearities for each excitation case. Because of the fundamental similarity between primary and superharmonic resonances, the quadratic and cubic nonlinearities were identical in both cases. On the other hand, there was a marked difference between them and the nonlinearities identified for subharmonic resonance. While this is an obvious shortcoming of our technique, we believe that the model simplicity justifies the added effort.

We found that it was necessary to include process noise in the model to capture the resonant (large-amplitude) response of the actuator around the peaks and measurement noise to capture small-amplitude motions (away from the peaks). We emphasize that the introduction of noise did not result in the appearance of new features (peaks) in the frequency spectrum. Its effects were limited to raising the power level of the resonant peaks and the simulated noise floor to match those measured experimentally. Process noise was almost constant across all three cases because the same experimental setup was employed. Likewise, the level of measurement noise was similar in all three cases because the excitation level was identical and motion sizes were similar.

Our parameter identification technique, in conjunction with the Duffing oscillator model, proved equally applicable to experimental FFTs obtained from primary, superharmonic, or subharmonic excitations, and reproduced the measured actuator response across all three cases. However, we found limitations in its applicability to secondary resonances. Specifically, it was not able to fully replicate the power levels in some of the response harmonics of secondary resonances. In addition, our identification technique should be extended to include flicker noise to enable it to more accurately capture low-frequency response.
